# Supplementary material for: Alcohol effects on globus pallidus connectivity: Role of impulsivity and binge drinking
Source: PLoS One. 2020 Mar 26;15(3):e0224906. doi: 10.1371/journal.pone.0224906 (PMC7098584; doi:10.1371/journal.pone.0224906)
Supplement: S1 File — Results and discussion of the main effects of alcohol on GPe connectivity. (PDF) [file pone.0224906.s001.pdf]

Alcohol effect on GPe connectivity

Using seed-to-voxel (i.e. whole brain) connectivity analyses - with GPe as seed region - provided the following significant results. Alcohol directly affected the GPe activity demonstrated by the shifting of the time series of the BOLD signal (Supplementary Figure 1A). See Supplementary Table 1 and Supplementary Figure 1B for seed-to-voxel connectivity results. These results are reported at a voxel-height threshold of $p<0.001$ and an extent threshold of $p<.05$ FDR corrected for multiple comparisons.

Right GPe connectivity changes

The seed-to-voxel functional connectivity revealed significant decreases in connectivity between the right GPe seed and the bilateral NAcc, bilateral putamen, subgenual anterior cingulate (ACC), bilateral caudate, and left orbitofrontal cortex (OFC) following IV alcohol infusion. Additional regions that significantly showed decreased connectivity were in the left cerebellum, right frontal pole and right middle frontal gyrus. We observed a significant alcohol-induced increase in connectivity between the right GPe seed and the right precentral gyrus, areas within the frontal pole, left OFC, inferior frontal gyrus (pars triangularis), left middle/superior temporal gyri (posterior portion), left occipital fusiform gyrus, and lateral occipital cortex (inferior division).

Left GPe connectivity changes

Reduced connectivity after alcohol infusion with the left GPe was detected in areas of the bilateral thalamus, bilateral caudate, right pallidum, and the left NAcc, putamen, and subgenual ACC/OFC. We also found significantly reduced left GPe connectivity with subregions of the frontal cortex (middle/superior gyri, right frontal pole, right central operculum, left precentral gyrus), cerebellum, precuneus, temporal cortex (left middle/superior gyri, left temporal pole, Heschl's gyrus), left angular gyrus, and left superior lateral occipital cortex. Alcohol-induced increases in connectivity with the left GPe were found in the bilateral paracingulate, medial frontal and superior frontal gyri, frontal pole, and insular cortices.

Supplementary discussion

We aimed to translate the preclinical finding from Abrahao et al. (2017) through an IV alcohol infusion paradigm. We measured resting-state functional connectivity in "sober" (i.e., BAC = 0.00 g/dl) and "binge drinking" (i.e., BAC = 0.08 g/dl) states. We did see functional connectivity between the right GPe and striatum regions decrease bilaterally in the "binge drinking" state, consistent with the rodent model. Given the theorized role of the GPe as part of the arkypallidal pathway, alcohol can be interpreted here as disrupting the ability of the GPe to send signals to stop or pause before actions [2].

In addition to the hypothesized dorsal striatum regions (i.e., caudate, putamen), we saw that alcohol reduced connectivity between the GPe and the ventral striatum (i.e., NAcc). This was unexpected given the lack of evidence for direct structural connectivity between these regions. However, there are several methodologic differences between work in animal and human models that may explain this finding. First, BOLD signal has low resolution in comparison to patch-clamp recordings. Electrophysiology has been used to validate the basal ganglia circuitry within a BOLD connectivity context using an optogenetic-resting state fMRI method [3], but given the GPe has common projections to the subthalamic nucleus with the ventral pallidum [4, 5], which we would expect to functionally connect with the NAcc, it is possible that connectivity signal from that region is confounding the GPe connectivity signal in this study. Moreover, resting state functional connectivity is an indirect measure of pathway communication and is unable to use timing to establish directionality. Thus, it is possible this finding may reflect indirect connectivity, such as through the ventral tegmental area [6].

We also unexpectedly found that alcohol infusion decreased connectivity between GPe and cerebellar / frontal pole areas, and increased connectivity between GPe and PFC / temporal gyri. These are not regions found to be directly connected to the GPe and involved in stop-signaling. However, as highlighted in the previous paragraph, these findings may reflect indirect connectivity. For example, cerebellar regions are connected to the CM/Pf complex of the thalamus [7], which is thought to be affected by GPe activity [5]. Our finding that alcohol increased connectivity between GPe and PFCl/temporal areas is particularly unexpected. However, previous studies have shown that substance dependent individuals have increased connectivity during rest in executive control networks [8] and between NAcc and dlPFC regions [9]. Given that resting state is typically associated with reduced executive control function, this increased coupling between GPe and lateral PFC activity may reflect impaired functioning. One possible mechanism underlying this finding could be that alcohol is impacting the function of long-range GABAergic projections between the GPe and frontal gyrus [10].

Our connectivity findings followed a somewhat lateralized pattern, where connections to left side regions from both right and left GPe increased with alcohol administration and connections to right side regions decreased. Alcohol has previously been shown to reduce the lateralization of specific functions, particularly in terms of greater left lateral increases and right lateral decreases in connectivity at rest [11]. The greater left-side increases in connectivity (both from ipsi- and contralateral GPe regions) may reflect this pattern as well. We would point out that there were mostly contralateral alcohol-decreases in connectivity from the left GPe, suggesting reductions in connectivity on the right side. We would also point out from the perspective of the role of the GPe pathways, previous work finds that connectivity strength in right-lateralized hyperdirect and indirect basal ganglia/frontal pathways predicted successful response inhibition (Jahfari et al., 2011). On the other hand, increases in left ipsilateral connectivity in individuals with chronic alcohol use has been associated with

compensatory function [12]. Taken together, the lateralization pattern may reflect alcohol related impairment of standard inhibitory pathways and increases in "alternative" neural communication routes.

Supplementary References

1. Abrahao KP, Chancey JH, Chan CS, Lovinger DM. Ethanol-Sensitive Pacemaker Neurons in the Mouse External Globus Pallidus. Neuropsychopharmacology : official publication of the American College of Neuropsychopharmacology. 2017;42(5):1070-81. doi: 10.1038/npp.2016.251. PubMed PMID: 27827370; PubMed Central PMCID: PMCPMC5506786.
2. Schmidt R, Leventhal DK, Mallet N, Chen F, Berke JD. Canceling actions involves a race between basal ganglia pathways. Nat Neurosci. 2013;16(8):1118-24. doi: 10.1038/nn.3456. PubMed PMID: 23852117; PubMed Central PMCID: PMCPMC3733500.
3. Bernal-Casas D, Lee HJ, Weitz AJ, Lee JH. Studying Brain Circuit Function with Dynamic Causal Modeling for Optogenetic fMRI. Neuron. 2017;93(3):522-32 e5. Epub 2017/01/31. doi: 10.1016/j.neuron.2016.12.035. PubMed PMID: 28132829; PubMed Central PMCID: PMCPMC5472443.
4. Root DH, Melendez RI, Zaborszky L, Napier TC. The ventral pallidum: Subregion-specific functional anatomy and roles in motivated behaviors. Progress in neurobiology. 2015;130:29-70.
5. Mastro KJ, Bouchard RS, Holt HA, Gittis AH. Transgenic mouse lines subdivide external segment of the globus pallidus (GPe) neurons and reveal distinct GPe output pathways. J Neurosci. 2014;34(6):2087-99.
6. Hart G, Leung BK, Balleine BW. Dorsal and ventral streams: the distinct role of striatal subregions in the acquisition and performance of goal-directed actions. Neurobiology of learning and memory. 2014;108:104-18.
7. Parent A. Extrinsic connections of the basal ganglia. Trends in neurosciences. 1990;13(7):254-8.
8. Krmpotich TD, Tregellas JR, Thompson LL, Banich MT, Klenk AM, Tanabe JL. Resting-state activity in the left executive control network is associated with behavioral approach and is increased in substance dependence. Drug and alcohol dependence. 2013;129(1-2):1-7.
9. Camchong J, Stenger A, Fein G. Resting-state synchrony in long-term abstinent alcoholics. Alcoholism: Clinical and Experimental Research. 2013;37(1):75-85.
10. Saunders A, Oldenburg IA, Berezovskii VK, Johnson CA, Kingery ND, Elliott HL, et al. A direct GABAergic output from the basal ganglia to frontal cortex. Nature. 2015;521(7550):85-9. Epub 2015/03/06. doi: 10.1038/nature14179. PubMed PMID: 25739505; PubMed Central PMCID: PMCPMC4425585.
11. Volkow ND, Ma Y, Zhu W, Fowler JS, Li J, Rao M, et al. Moderate doses of alcohol disrupt the functional organization of the human brain. Psychiatry Research: Neuroimaging. 2008;162(3):205-13. doi: https://doi.org/10.1016/j.pscychresns.2007.04.010.
12. Chanraud S, Pitel AL, Pfefferbaum A, Sullivan EV. Disruption of Functional Connectivity of the Default-Mode Network in Alcoholism. Cereb Cortex. 2011;21(10):2272-81. doi: 10.1093/cercor/bhq297. PubMed PMID: WOS:000294808800007.
