# Supplementary figures and images for: Alcohol effects on globus pallidus connectivity: Role of impulsivity and binge drinking
Source: PLoS One. 2020 Mar 26;15(3):e0224906. doi: 10.1371/journal.pone.0224906 (PMC7098584; doi:10.1371/journal.pone.0224906)

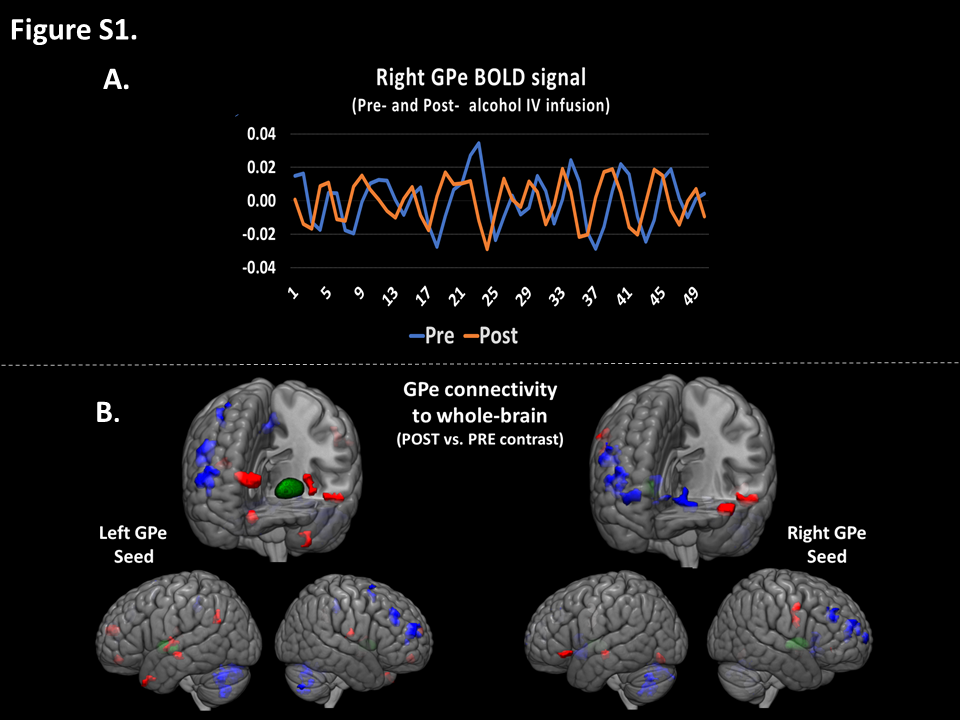

Supplement: S1 Fig — (A) Shows the first 50 time points of the timeseries of the preprocessed BOLD signal from the right GPe (averaged across voxels within the right GPe and across subjects; sampled every two seconds), before (pre) and after (post) the IV-alcohol infusion. (B) Change in the GPe whole brain connectivity (Post vs. Pre contrast). Regions that decreased connectivity with the GPe after IV-alcohol infusion are in blue; regions with increased connectivity are in red; GPe masks are in green. (TIF) [file pone.0224906.s002.tif]
