# Supplementary material for: Alcohol effects on globus pallidus connectivity: Role of impulsivity and binge drinking
Source: PLoS One. 2020 Mar 26;15(3):e0224906. doi: 10.1371/journal.pone.0224906 (PMC7098584; doi:10.1371/journal.pone.0224906)
Supplement: Attachment — Submitted filename: Fede_etal_PLOS-ONE_Revision-Response.docx [file pone.0224906.s005.docx]

**Editor Comment:**.

A residual point that needs to be addressed in the discussion section is the interpretation of reduced behavioral inhibition in changed GPe pathway functioning in individuals with more drinking. I agree with reviewer 2, that without the support of behavioral data in the current data set, this interpretation indeed should be toned down.
**Reviewer Comment:**

The authors did a good job in addressing most of my concerns. Without behavioral measurements, the data of this study are not supporting the discussion that changes in GPe pathways in individuals with more drinking are associated with reduced behavioral inhibition. The response of the authors that they were testing alcohol-related changes in GPe pathways in a human model should have enabled them to apply a response inhibition task, if that was one of the main hypothesis of the study. This part of the discussion needs to be toned down or clearly noted as a limitation of the study.

**Response:**

We have revised our discussion throughout to soften the interpretation of reduced behavioral inhibition. As such, we have added alternative mechanisms that may explain our findings throughout the Discussion. We have also added to the last paragraph of the limitations section (line 452/453) as follows: “Because we do not have behavioral data on response inhibition in the alcohol intoxication state, there are other potential explanations for our findings.”
